# Quantitative analysis of serum metabolites in a rat model of Alzheimer’s disease

**DOI:** 10.3389/fnagi.2025.1648561

**Published:** 2025-09-23

**Authors:** Anton A. Smolentsev, Darya V. Telegina, Nataliya G. Kolosova, Yuri P. Tsentalovich, Olga A. Snytnikova

**Affiliations:** ^1^International Tomography Center, Siberian Branch of the Russian Academy of Sciences, Novosibirsk, Russia; ^2^Department of Natural Sciences, Novosibirsk State University, Novosibirsk, Russia; ^3^The Federal Research Center Institute of Cytology and Genetics, Siberian Branch of the Russian Academy of Sciences, Novosibirsk, Russia

**Keywords:** Alzheimer’s disease, aging, OXYS rats, serum, metabolomic profile

## Abstract

**Objective:**

OXYS rats are a unique animal model of sporadic Alzheimer’s disease (AD) that demonstrates all the key signs of AD in humans. Studying metabolic processes in OXYS rats in comparison with control Wistar rats can contribute to understanding the mechanisms of AD development, as well as to establishing metabolomic biomarkers of AD. The main goals of the work are to establish differences in the metabolomic profiles of OXYS and Wistar rat serum at different stages of AD-like pathology (presymptomatic, early and late).

**Methods:**

NMR-based metabolomics was applied for metabolomic profiling of blood serum of OXYS and Wistar rats at the age of 20 days (presymptomatic period), 4 months (first manifestation of signs of AD) and 16 months (active development of signs).

**Results:**

We determined the concentrations of 55 metabolites present in rat serum. We found that age-related changes in both rat strains reflect animal maturation (20 days to 4 months) and aging (4 months to 16 months), and correspond mainly to amino acid metabolism, purine metabolism, and energy pathways. Potential AD blood biomarkers include lysine, BCAAs, alanine, ornithine, creatine, glutamine and pyruvate.

**Conclusion:**

The most significant differences between OXYS and Wistar blood metabolomes were found for 20-day-old animals, which corresponds to the preclinical period of AD development in humans. Metabolomic changes observed in the brain and blood are different and often opposite in sign. Blood serum is potentially promising fluid for AD diagnosis.

## 1 Introduction

Alzheimer’s disease (AD) is the most common neurodegenerative disorder and the leading cause of senile dementia that have no effective treatment. Age is the most significant risk factor for sporadic form of disease (more than 95% of cases), in which the first signs of cognitive impairment appear over the age of 65 years against the background of irreversible neurodegenerative changes that develop asymptomatically over many years. As life expectancy increases, by 2050 the number of people worldwide with dementia will exceed 150 million (Alzheimer's Association Report, 2023). Alzheimer’s disease is characterized by abnormal deposition of beta-amyloid (Aβ), hyperphosphorylated tau proteins, and mitochondrial dysfunction in the brain. All this, together with such phenomena as reactive astrogliosis and neurodegeneration, contributes to the occurrence of cognitive impairment and ultimately leads to dementia (Swerdlow, 2020). The need to prevent these processes by taking measures aimed at combating AD at the earliest stages of its development is actively discussed (Ritchie et al., 2017). Research over the last decade using new technologies has shown that the preclinical period of AD can last for decades. The question of the initial moment of development of AD disease and what contributes to it remains still open. There is a pressing need for early diagnosis of AD which is driving the search of new approaches. Neuroimaging and cerebrospinal fluid biomarkers could help detect and diagnose the disease, but unfortunately, the clinical implementation of these methods is limited by their availability, cost, and invasiveness. Therefore, the search for blood-based biomarkers that would allow earlier and faster diagnosis, and could also help in risk assessment, early detection, prognosis, and treatment of AD is currently becoming an urgent task. However, a recent compelling international study has shown that assessing plasma levels of the traditional AD markers amyloid and phospho–tau can only confirm doctors’ conclusions, increasing their confidence in the diagnosis, but have a little impact on its establishing (Altomare et al., 2024). It can be assumed that in the future, such plasma biomarkers could significantly improve the efficiency of monitoring AD progression or treatment response, but are unlikely to be useful as early markers of the disease, unlike metabolomic markers (González-Domínguez et al., 2015; Weng et al., 2022; Nielsen et al., 2024).

Metabolic dysfunction is one of the risk factors for AD, which appears to contribute or perhaps even trigger pathological molecular cascades of AD-pathology in conjunction with changes in various metabolites in the body. It is noteworthy, that a metabolomic analysis of blood in amyloid-positive individuals identified metabolites the levels of which differed between individuals with progressive cognitive decline and those whose cognitive abilities remained unchanged 3 years before cognitive decline (Tremblay-Franco et al., 2024). It is suggested that pathological changes in the brain may be reflected in blood metabolites which are expected to be candidate biomarkers.

However, it is unclear to what extent changes in the blood metabolome reflect changes in the brain at the preclinical stage of AD. Research progress in this direction can be facilitated by the use of quantitative metabolomics methods, a high-throughput technology that allows for the simultaneous detection and cataloging of a large number of metabolites, which can be a useful tool in studying the pathogenesis of the disease, identifying its predictors and biomarkers that reflect the rate of AD progression and response to therapy (Dong and Brewer, 2019; Huo et al., 2020; Niedzwiecki et al., 2020; Ozaki et al., 2022; Jaramillo-Jimenez et al., 2023; Judd et al., 2023; Pandey et al., 2024). At the same time, the study of preclinical stages of the disease in humans, the manifestation of which occurs later than the formation of neurodegenerative changes and the development of deep events at the molecular level, is problematic. The aim of this study is to compare changes in serum metabolomes in the dynamic of AD-like pathology development in senescence-accelerated OXYS rats, a unique model of the sporadic form of the disease. These animals spontaneously develop all the main hallmarks of Alzheimer’s disease: structural neurodegenerative changes, neuronal loss, synaptic failure, disturbances in neurotrophic supply, cerebrovascular changes, accumulation of Aβ_1–42_ and hyperphosphorylation of tau protein in the hippocampus, as well as cognitive impairment against the background of increasing mitochondrial dysfunction with age (Kolosova et al., 2014, 2022; Stefanova et al., 2014). In our recent work (Snytnikova et al., 2024), we studied the metabolomic composition of the hippocampus of OXYS and Wistar rats and found sets of differential metabolites for different ages. In particular, we found increased accumulation of scyllo-inositol and decreased hypotaurine content in the brain of OXYS rats compared to Wistar rats. In this work, we studied the serum metabolome of OXYS rats at different stages of development of pathology similar to Alzheimer’s disease, including presymptomatic stage, using Wistar rats as a control. The results of the study were compared with data on the hippocampal metabolome of these animals (Snytnikova et al., 2024).

## 2 Materials and methods

### 2.1 Chemicals

All reagents were purchased from Sigma-Aldrich (St. Louis, Michigan, USA) with the following exceptions: phosphate-buffered saline was purchased from Biolot (Moscow, Russia), D_2_O 99.9% and sodium 4,4-dimethyl-4-silapentane-1-sulfonate (DSS) were purchased from Cambridge Isotope Laboratories Inc. (Tewkesbury, Massachusetts, USA). Water was deionized to a quality of 18.2 MΩ using an ultrapure water system (SG water, Munich, Germany).

### 2.2 Animals

In this work, we used the genetic model of senescence-accelerated OXYS rats. This strain is characterized by accelerated aging and spontaneous development of all the key features of Alzheimer’s disease. OXYS rats develop the manifestation of behavioral alterations and learning and memory deficits at the same time with the hyperphosphorylation of the tau protein in the hippocampus and cortex, impaired long-term potentiation and first signs of neurodegeneration at about 3–5 months. With age, neurodegenerative changes in the brain of OXYS rats become amplified against the background of overproduction of amyloid precursor protein (A*β*PP), accumulation of β-amyloid, and by the age of 16–18 months reach the well-pronounced stages of the AD-like pathology (Kolosova et al., 2014). The study was performed using rat males from three age groups: age 20 days, which corresponds to the “presymptomatic” period without the development of AD signs; 4 months - with manifestations of signs, and 16 months with the active progression of AD signs (“early” and “late” stages, respectively) (Kolosova et al., 2014; Stefanova et al., 2014). Age-matched Wistar rats were used as control. The keeping of animals as described in Snytnikova et al. (2024) and all experiments with animals were carried out in accordance with the regulation on the ethics of using animals in research supported by the Russian Science Foundation,^1^ as well as with the European Union Directive 2010/63/EU and approved by the Commission on Bioethics at the Institute of Cytology and Genetics of the Siberian Branch of the Russian Academy of Sciences (Protocol no. 85/1 dated June 18, 2021).

### 2.3 Sample collection, metabolite extraction and NMR spectroscopy

Euthanasia of the animal was performed by introducing 100% carbon dioxide into an unlined cage initially containing room air with the lid closed at a rate sufficient to produce rapid anesthesia. Death occurred within 1 min. The rat was immediately decapitated and blood was collected from the body. To prepare the serum, the blood was allowed to clot for 15 min and then centrifuged for 15 min at 3000 g. The supernatant was collected and frozen in liquid nitrogen. All collected serum samples were stored at −70 °C until analyzed.

The extraction of water-soluble metabolites from the rat blood serum was performed by the sample preparation protocol earlier evaluated in our lab (Snytnikova et al., 2022). A cold methanol-chloroform extraction was chosen as the most efficient method for extracting metabolites from blood serum. The volumes of serum and extracting solution were used at the ratio: serum/methanol/chloroform = 1/1/1. In this work, 300 μL of cold methanol (−20 °C) and 300 μL of cold chloroform (−20 °C) were added to 300 μL of serum. Samples were mixed on a mini-vortex centrifuge and placed in a shaker for 30 min at 1300 rpm. The samples were kept in a freezer at −20 °C for 30 min, then centrifuged at 12,000 rpm at 4 °C for 30 min, and the supernatant was taken. The top hydrophilic fraction was collected and dried on a vacuum evaporator. The dried extracts were dissolved in 600 μL deuterated phosphate buffer (0.01 M, pH = 7.4) containing 6 μM DSS as an internal standard.

The metabolomic composition of the obtained extracts was analyzed by the method of nuclear magnetic resonance using an AVANCE III HD 700 MHz NMR spectrometer (Bruker BioSpin, Ettlingen, Germany) at the Center of Collective Use “Mass spectrometric investigations” SB RAS. The ^1^H spectra were recorded using the 5 mm TXI ^1^H-^13^C/^15^N/D ZGR probehead. The spectra were acquired at using a single pulse zgpr sequence (detection pulse was 90 degrees) with the water signal suppression (saturation of the water signal with low power (20 μW) continuous RF during the delay between repetitions), 14 ppm spectral width, 5 s relaxation delay, 6.7 s acquisition time. For each sample, the total spectrum of the sample was obtained by the sum of the 64 accumulated spectra. The temperature of the sample during the recording of the spectrum was maintained at 25.0 ± 0.1 °C, the magnetic field homogeneity was shimmed with a Topshim procedure, and the sample volume in 5 mm NMR tubes was 0.6 mL. The parameters of the spectra recording were the same as described in the works (Glinskikh et al., 2021; Snytnikova et al., 2022, 2024). The chemical shift of metabolites were determined relatively DSS (chemical shift 0.00 ppm). The assignment of the metabolite resonances was carried out by comparing the obtained data with the data in Human Metabolome Database^1^ and Wishart et al. (2022), or by adding reference compounds whenever needed, and also based on our own experience in the metabolomic profiling (Snytnikova et al., 2017, 2019, 2022, 2024; Glinskikh et al., 2021). We compared the resonances of individual substances with the resonances observed in the spectrum of the sample, which is actually a superposition of the spectra of individual substances. The concentrations of the metabolite were determined by signals which do not overlap in the spectrum with signals from other metabolites. To determine the metabolite concentrations we used the same metabolite list with resonances published earlier in the Supplementary material of study (Snytnikova et al., 2022). The phases and baselines of the collected spectra were manually corrected using MestReNova V.12 (Mestrelab Research S. L.) software. The absolute concentrations of metabolites in the samples were calculated by the integration of the peak area of the metabolite signals relatively to the DSS signal. A detailed description of the concentration determination was published in the works (Glinskikh et al., 2021; Snytnikova et al., 2022).

### 2.4 Analysis of metabolite concentrations

The normalized metabolite concentration data were used to reveal the general metabolomic differences. PCA and sPLS-DA were performed using autoscaled (mean-centered and divided by the standard deviation of each variable) quantitative metabolomic data. To analyze the contributions of all metabolites into metabolic fingerprints we apply the principal component analysis (PCA) and sparse partial least squares discriminant analysis (sPLS-DA). To determine the meaningful patterns of metabolite concentration differences, the Metabolite Set Enrichment Analysis (MSEA) and Metabolite Pathway Analysis (MetPA) using the Kyoto Encyclopedia of Genes and Genomes database [KEGG; (Kanehisa et al., 2014)] were applied. These analyzes were performed using the MetaboAnalyst 6.0 web-platform MetaboAnalyst^1^ (Pang et al., 2021). The Statistical Analysis (one factor) module was used to construct the PCA scores and Volcano plots. The MSEA plots were constructed with the Enrichment Analysis module.

### 2.5 Statistical analysis

The obtained data of metabolite concentration were analyzed by one-way and two-way analysis of variance (ANOVA) using the Google Colaboratory^1^ and the Python library of the Numpy and Scipy Documentation^1^, graphs were built using the Matplotlib: Visualization with Python^1^. The Shapiro–Wilk test was used to check the normality of distributions. The Levene’s test was used to assess the homogeneity of dispersions. *Post hoc* multiple comparisons test was used with ANOVA analysis of variance. The genotype (Wistar, OXYS) and age of the animals (20 days, 4 months, and 16 months) were considered as independent factors in two-way ANOVA. The results were considered statistically significant at *p* < 0.05 with FDR adjustment. Statistical calculation values are presented in Supplementary information.

## 3 Results

### 3.1 Quantitative metabolomic profiling of serum

The performed metabolomic profiling of rat serum revealed a total of 55 metabolites. For each sample group studied, the range of variation and the average value of the metabolite concentrations were determined. The determined concentrations of metabolites ranged from 0.3 μM to 7.6 mM. The most abundant metabolites in the blood serum (the concentration above 300 μM) were lactate, glucose, alanine, glutamine, 3-hydroxybutyrate, glycine, creatine and lysine. Table 1 shows the concentrations of metabolites with the most significant differences between rat strains. Tables with concentrations of all metabolites measured in this work are presented in the Supplementary Table S1—Wistar rats, Supplementary Table S2—OXYS rats.

**Table 1:** Concentrations of rat serum metabolites with the most significant interstrain differences (indicated by asterisk).

Metabolite	20 days		4 months		16 months	
	Wistar	OXYS	Wistar	OXYS	Wistar	OXYS
2-Ketoisovalerate	4.1 ± 2.5	5.6 ± 1.0	4.4 ± 0.9	4.0 ± 0.9	7.9 ± 0.9	4.9 ± 1.3^*^
3-Methyl-2-oxovalerate	14 ± 5	11.2 ± 2.3	10.6 ± 1.8	7.1 ± 1.2^*^	15 ± 4	7.4 ± 2.5^*^
Allantoin	39 ± 9	52 ± 7^*^	73 ± 10	64 ± 5	50 ± 7	51 ± 6
Ascorbate	0.9 ± 0.4	1.4 ± 0.4	19 ± 7	20 ± 7	5.8 ± 2.9	15.9 ± 2.0^*^
Citrate	280 ± 40	270 ± 40	238 ± 28	174 ± 21^*^	210 ± 50	180 ± 40
Creatine	180 ± 30	264 ± 15^*^	296 ± 30	330 ± 40	350 ± 40	360 ± 50
Cytidine	35 ± 8	35 ± 12	68 ± 8	58.0 ± 1.5^*^	60 ± 7	61 ± 6
Glutamine	600 ± 70	490 ± 60^*^	550 ± 70	540 ± 50	750 ± 90	670 ± 60
Glycerol	135 ± 22	160 ± 30	120 ± 40	120 ± 70	143 ± 29	77 ± 16^*^
Glycine	219 ± 16	280 ± 40^*^	290 ± 40	315 ± 24	330 ± 50	310 ± 30
Histidine	45 ± 7	60 ± 7^*^	60 ± 11	67 ± 3	68 ± 9	70 ± 6
Isoleucine	84 ± 13	63 ± 10^*^	82 ± 13	88 ± 9	101 ± 15	99 ± 16
Leucine	140 ± 30	103 ± 11^*^	133 ± 21	151 ± 25	151 ± 23	151 ± 22
Lysine	127 ± 25	250 ± 80^*^	240 ± 40	245 ± 29	310 ± 30	270 ± 40
Mannose	22.5 ± 2.6	29 ± 4^*^	33 ± 10	31 ± 6	30 ± 3	31 ± 3
Proline	180 ± 70	164 ± 19	164 ± 29	140 ± 10	190 ± 40	131 ± 21^*^
Sarcosine	4.2 ± 1.9	4.4 ± 0.8	1.97 ± 0.19	3.1 ± 0.6^*^	2.7 ± 0.5	3.4 ± 1.1
Threonine	160 ± 50	182 ± 21	230 ± 40	219 ± 18	350 ± 90	210 ± 30^*^

Data presented in μM as Mean ± SD.

### 3.2 Age-related changes in the metabolomic profiles

**Figure 1:**
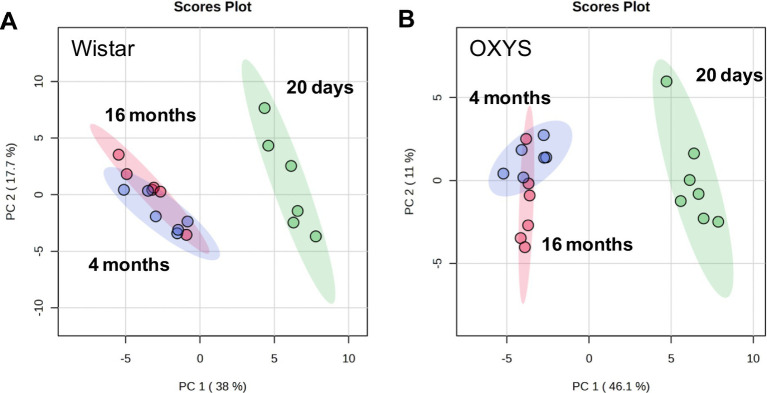
Principal component analysis of metabolomic profile of blood serum from Wistar **(A)** and OXYS **(B)** rats of various ages. Colored ovals indicate 95% confidence regions.

Age-related changes in the serum metabolome were evaluated based on the score values of multivariate principal component analysis (PCA). A comparison was made of age-related changes in the blood metabolomic profiles during physiological aging of Wistar rats and senescence-accelerated OXYS rats. Age-related differences for Wistar and OXYS rats in terms of the totality of all metabolites are shown in Figure 1 by PCA scores plots, where the number of points corresponds to the number of studied samples in a group.

For both Wistar and OXYS rats, the serum metabolomic profile at 20 days of age differs significantly from that of 4- and 16-month-old animals. The separation of the sample populations depending on age occurs mainly along the first principal component (38.0% for Wistar and 46.1% for OXYS). The group difference is best visualized by the sPLS–DA plot presented in Supplementary Figure S1, where the separation between 4- and 16-month-old animals occurs along the second component. Age-related changes for all 55 metabolites detected in this work are shown in Supplementary Figure S2, and statistical analysis of age-related changes is presented in Supplementary Tables S3, S4.

To analyze age-related changes in rat serum metabolomic profiles in more detail, we constructed PCA score plots, Volcano plots, and performed MSEA for 20 days and 4 months of age, and separately for 4 months and 16 months of age. The analyses were performed for both Wistar and OXYS rats (Supplementary Figures S3.1–S3.4). Since age-related differences were significant, we reduced the number of differential metabolites identified by using relatively high cutoff values: fold change above 1.3, *p*-value below 0.05. This way, we identified 20 differential metabolites (concentration of 8 compounds decreased and 12 compounds increased) when comparing 20-day-old and 4-month-old Wistar, 32 differential metabolites (19 decreased and 13 increased) when comparing 20-days and 4-months OXYS rats (Supplementary Figures S3.1 and S3.2, respectively; Supplementary Table S3). In the comparison of 4-month-old and 16-month-old animals, the list of significantly different metabolites is much shorter: 14 compounds (5 decreased and 9 increased) for Wistar and only 2 metabolites (1 decreased and 1 increased) for OXYS (Supplementary Figures S3.3 and S3.4, respectively; Supplementary Table S3).

**Figure 2:**
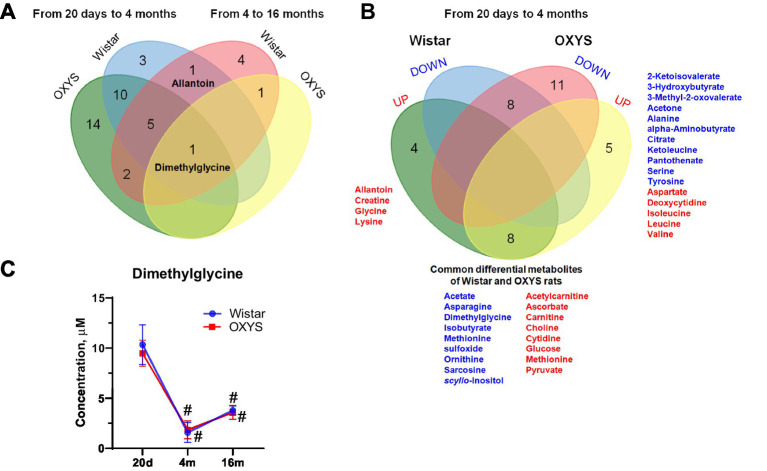
Comparison of age-related changes of metabolite concentrations (Fold Change > 1.3, *p*-value < 0.05) in OXYS and Wistar rats using a Venn diagram: overall changes from 20 days to 4 months and from 4 months to 16 months **(A)**; detailed analysis of data from 20 days to 4 months **(B)** (red and blue indicate increased and decreased concentration, respectively). **(C)** Serum dimethylglycine concentrations in Wistar and OXYS rats; data are presented as mean ± SD. Significant differences: # *p*-value < 0.05 with previous age. The sign 20d in the figure corresponds to the age of 20 days, 4 m and 16 m - 4 and 16 months, respectively.

Venn diagram (Figure 2A) demonstrates the metabolic shift at 20 days versus 4 months of age and at 4 months versus 16 months of age in Wistar and OXYS rats. Since metabolomic changes between 20 days and 4 months are more significant, we also constructed a detailed Venn diagram for these ages, which indicates the directions of metabolomic changes (Figure 2B). Figure 2B also contains a list of differential metabolites common for both OXYS and Wistar rats. Figure 2C presents an example of metabolite concentration changes with age in the rat serum: the level of dimethylglycine decreases between 20 days and 4 months of age, and then increases for both OXYS and Wistar rats. In addition to dimethylglycine, codirectional changes were found for 15 metabolites from 20 days to 4 months of age (8 decreased and 8 increased, as shown in Figure 2B) and a decrease in phosphocholine concentration from 4 months to 16 months of age in both Wistar and OXYS rats (Supplementary Tables S1, S2). In the age period from 20 days to 4 months, an increase in the concentration of allantoin, creatine, glycine and lysine was observed specifically for Wistar rats, whereas in OXYS rats there was a change in the concentration of 16 metabolites (Figure 2B). In the period from 4 to 16 months of age, the concentration of dimethylglycine increased and phosphocholine decreased in the blood serum of rats of both lines. The concentration of methionine increased unidirectionally in all periods of the study and the concentration of scyllo-inositol decreased in Wistar rats (Supplementary Table S1). At the same time, the level of 5 metabolites in the blood serum of Wistar rats changed in different directions: the concentration of allantoin and ascorbate increased at the age of 4 months and decreased at the age of 16 months, whereas dimethylglycine, methionine sulfoxide and sarcosine demonstrated opposite directions of concentration change (Supplementary Table S1). From 4 to 16 months, the levels of 6 metabolites changed specifically for Wistar rats, of which only the concentration of betaine decreased, whereas the concentration of 2-ketoisovalerate, arginine, glutamine, ketoleucine and threonine increased (Supplementary Table S1).

### 3.3 The effect of the AD-like pathology on the serum metabolome

**Figure 3:**
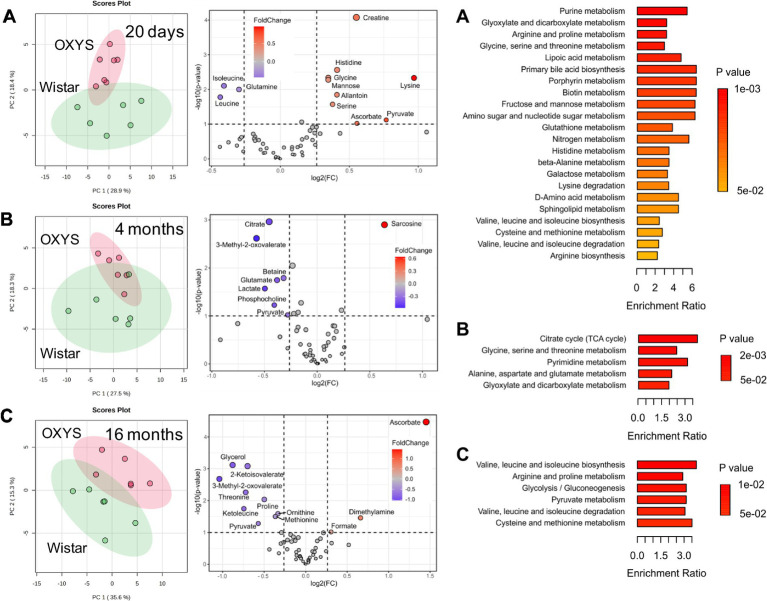
PCA scores plots (left), Volcano plots (middle) and enriched metabolite sets (p-value < 0.05) for serum metabolites from age-matched Wistar and OXYS rats at different ages: 20 days **(A)**, 4 months **(B)**, 16 months **(C)**. In Volcano plots, the *x*-axis displays the fold change (FC), the horizontal line depicts the cut-off of *p*-value = 0.1.

**Figure 4:**
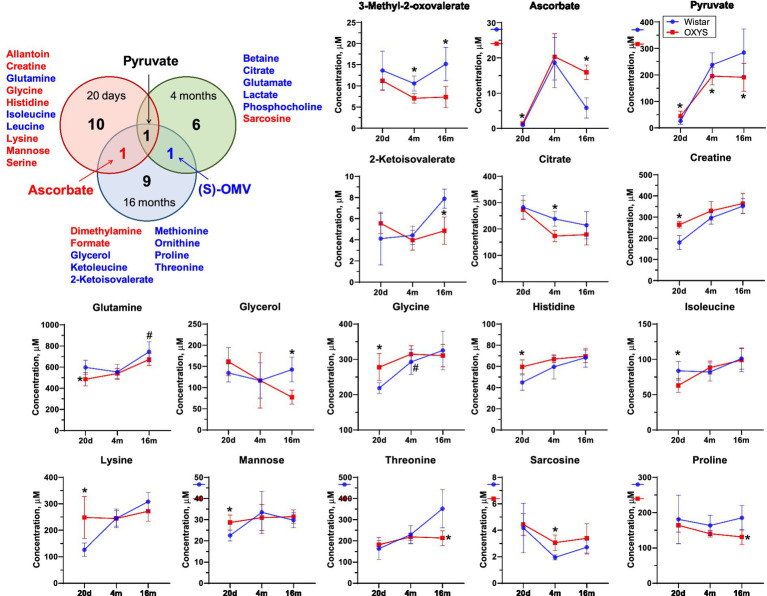
Venn diagram for differential metabolites in blood of OXYS and Wistar rats at 20 days, 4 months, and 16 months. Red and blue letter indicate increased and decreased concentration, respectively. Comparison of metabolite concentrations in Wistar (blue circle) and OXYS (red square) rat serum during the development of AD signs: presymptomatic (at 20 days of age), early (4 months) and late (16 months) stages. Data are presented as mean ± SD. Significant differences: *p-value < 0.05 between rat strains. (S)-OMV, 3-Methyl-2-oxovalerate. The sign 20d on the x-axis displays data corresponding to the age of 20 days, 4 m and 16 m - 4 and 16 months, respectively.

To assess metabolome changes as AD develops and progresses, we compared serum metabolome profiles of age-matched Wistar and OXYS rats (Figure 3). PCA demonstrates that interstrain differences are greatest at 20 days of age, when AD signs have not yet been detected in OXYS rats, and are lowest during the period of active AD manifestation at 4 months of age. In 20-day-old OXYS rats, the levels of three metabolites (leucine, isoleucine and glutamine) are reduced and the levels of nine metabolites (creatine, histidine, glycine, mannose, allantoin, serine, lysine, pyruvate and ascorbate) are increased compared to Wistar rats. In 4-month-old OXYS rats, the levels of citrate, 3-methyl-2-oxovalerate, betaine, glutamate, lactate, phosphocholine, and pyruvate are decreased, and there is only one increased metabolite, sarcosine. At the age of 16 months, the levels of glycerol, 2-ketoisovalerate, 3-methyl-2-oxovalerate, threonine, proline, ketoleucine, ornithine, methionine, and pyruvate are reduced in OXYS rats, while the levels of ascorbate, dimethylamine, and formate are increased compared to Wistar rats. The graphs in Figure 4 show examples of metabolites with the greatest differences between age-matched OXYS and Wistar rats.

We found three metabolites the concentrations of which changed significantly for at least two ages. The concentration of pyruvate was increased in OXYS rats at 20 days of age, and decreased at 4 months and 16 months of age. Serum ascorbate levels were significantly higher in OXYS rats than in Wistar rats at 20 days and 16 months of age and were similar at 4 months of age. The metabolite 3-methyl-2-oxovalerate was significantly lower in OXYS at 4 months and 16 months of age.

MSEA performed for age-matched OXYS and Wistar rats indicates metabolic pathways with the largest and statistically significant differences between the strains (Figure 3; Supplementary Tables S5–S7). At the age of 20 days, 22 pathways comprising 31 metabolites detected in this work were statistically different with *p* < 0.05. For 4-month-old rats, a significant difference was found for five pathways (18 metabolites), and for 16-month-old animals for six pathways (15 metabolites). For 20 days and 4 months of ages, two pathways were common. These pathways are “Glycine, serine and threonine metabolism,” which includes the metabolites serine, choline, betaine, dimethylglycine, glycine, sarcosine, threonine, creatine, and pyruvate, and “Glyoxylate and dicarboxylate metabolism,” which includes citrate, serine, glycine, acetate, pyruvate, formate, and glutamine. For 20 days and 16 months of ages, the common pathways are “Arginine and proline metabolites,” “Valine, leucine and isoleucine biosynthesis,” and “Valine, leucine and isoleucine degradation.” None of metabolic pathways found at 16 months of age matched those observed in 4-month-old animals.

## 4 Discussion

The first aim of our study was to investigate the changes in the serum metabolome during aging and the development of AD signs in rats and, secondly, to validate blood-based biomarkers to aid in AD diagnosis, especially at the early, presymptomatic stage. To gain deeper insight into the pathophysiology of dementia and to identify new potential diagnostic serum markers for AD, we analyzed the serum metabolome profile of Wistar and OXYS rats at different stages of AD signs development and compared these data with previously obtained metabolomic profiles in the rat hippocampus (Snytnikova et al., 2024). The main function of blood is to deliver nutrients and remove waste products, so it is important to recognize that different levels of metabolites in the blood may result from different biological processes occurring in the body. Thus, with the development of a pathological process, an increase in a particular metabolite may be observed, but at the same time, overproduction of this compound may reflect the physiological response of the body to suppress the disease. Although elucidating metabolic mechanisms is a complex task, this does not prevent their use for clinical assessments, thus bringing practical benefits (Weng et al., 2022).

In rat serum, the greatest age-related differences in the metabolic profile were found between 20 days and 4 months of age, while the difference between 4 months and 16 months was less pronounced (Figure 1; Supplementary Figures S1, S3.1–S3.4). The differential metabolite lists in Wistar and OXYS rats between 20 days and 4 months of age were similar (Supplementary Table S3; Supplementary Figures S3.1, S3.2). MSEA showed that the most affected metabolic pathways during this time period were “Alanine, aspartate, and glutamate metabolism,” “Cysteine and methionine metabolism,” “Glycine, serine, and threonine metabolism,” “Glycolysis/gluconeogenesis,” and “Pyruvate metabolism” for both rat strains (Supplementary Figures S3.1, S3.2). Apparently, these age-related metabolomic changes observed in both Wistar and OXYS rats appear to be related to the maturation of the animals. The metabolomic changes reflecting animal aging (4 months vs. 16 months) and common to both strains correspond to “Purine metabolism,” “Nitrogen metabolism” and “Arginine biosynthesis” (Supplementary Figures S3.3, S3.4). Metabolomic studies in other animal models have also demonstrated age-related changes in similar metabolites and associated pathways (Yin et al., 2023). Interestingly, we found only one metabolite, dimethylglycine, the level of which changes with aging in both rat strains (Figure 2C). This is an intermediate metabolite in the metabolism of choline to glycine that exhibits antioxidant activity, most likely through free radical scavenging with a minor contribution to the respiratory chain (Finsterer and Bindu, 2015). On the other hand, elevated plasma dimethylglycine levels have been shown to be associated with an increased risk of acute myocardial infarction and coronary heart disease (Wei and Wang, 2021). Thus, it can be assumed that the increase in dimethylglycine levels by 16 months in Wistar and OXYS rats reflects increasing vascular dysfunction, which is characteristic of both normal and accelerated aging.

To search for potential AD biomarkers in blood, we examined the interstrain differences in the serum metabolomic profiles during the development of AD signs in OXYS rats. According to modern studies (De-Paula et al., 2012), three clinical phases of AD can be defined: (1) pre-symptomatic (or preclinical) AD; (2) pre-dementia phase of AD (compatible with the definition of progressive, amnestic mild cognitive impairment); (3) phase of clinically defined dementia in AD. It is clear that there is no biological model that completely reproduces all the features of human AD. However, non-transgenic OXYS rats largely reproduce the stages of the disease (Stefanova et al., 2014, 2015; Rudnitskaya et al., 2021; Kolosova et al., 2022). The age of 20 days corresponds to the presymptomatic stage of AD, when OXYS rats do not show signs of the disease. At the age of 3–5 months, against the background of mitochondrial dysfunction OXYS rats manifest signs of AD such as hyperphosphorylation of tau-protein, disruption of long-term post-tetanic potentiation, synaptic deficiency, destructive neuron changes, behavioral disorders and reduction of cognitive functions. This stage we can define as the pre-dementia phase of AD. By 16 months, OXYS rats shows active progression of AD signs against the background of increased APP levels, enhanced accumulation of Aβ and formation of amyloid plaques in the brain, and a transition to the stage of disease occurs, which can be defined as an analogue of the dementia phase of AD in humans.

For 20-day-old rats, the highest and statistically significant interstrain differences were found for several amino acids, organic acids, and sugars (Figures 2, 4; Supplementary Table S3), and the most affected metabolic pathways are “Purine metabolism,” “Glyoxylate and dicarboxylate metabolism,” “Arginine and proline metabolism,” and “Glycine, serine and threonine metabolism” (Figure 3). This is in a good agreement with the results obtained for the human blood from AD patients (Trushina et al., 2013; Trushina and Mielke, 2014; Nielsen et al., 2021, 2024; Whiley et al., 2021; Weng et al., 2022; Berezhnoy et al., 2023; Yu et al., 2023). In particular, changes in glycolysis, the Krebs cycle, and the urea cycle in the human blood under AD development were found in study (Weng et al., 2022), while Nielsen et al. reported that the most affected pathways were “Purine metabolism,” “Metabolism of branched-chain amino acids,” “Glycolysis and gluconeogenesis,” and “Lysine metabolism” (Nielsen et al., 2021, 2024). In the work (Trushina et al., 2013), alternations in “Lysine metabolism,” “TCA cycle,” “Pyruvate metabolism,” and “Valine, leucine and isoleucine metabolism” were detected in the blood of AD patients.

An analysis of the literature data on human blood metabolomics related to Alzheimer’s disease shows that lysine is the metabolite most often proposed as a potential biomarker of AD (Trushina et al., 2013; Zetterberg and Burnham, 2019; Ozaki et al., 2022; Nielsen et al., 2024). Other potential biomarkers include BCAAs, alanine, ornithine, creatine, glutamine and pyruvate (Trushina et al., 2013; Kelley and Andersson, 2014; Zetterberg and Burnham, 2019; Nielsen et al., 2021, 2024; Ozaki et al., 2022; Weng et al., 2022; Berezhnoy et al., 2023; Yu et al., 2023). Most of these potential biomarkers were detected in the present work as differential metabolites between OXYS and Wistar rats. We found a decrease in the level of leucine, isoleucine and glutamine and an increase in the level of nine metabolites (creatine, histidine, glycine, mannose, allantoin, serine, lysine, pyruvate and ascorbate) in 20-day-old OXYS rats compared to Wistar rats. The most statistically significant difference is observed for creatine, the concentration of which in the blood serum of OXYS rats is higher, 260 μM versus 180 μM in Wistar. Creatine is a nitrogen-containing carboxylic acid involved in metabolic pathways of energy metabolism, and it is an important source of energy for the body. Elevated levels of this metabolite may indicate disturbances in guanidinoacetate metabolism (Berezhnoy et al., 2023), as well as disturbances in the Krebs cycle and modification of creatine kinase enzyme (Castegna et al., 2002; Wang et al., 2020). In a recent study, creatine was proposed as one of the biomarkers for diagnosing Alzheimer’s disease in humans (Nielsen et al., 2024). It should also be noted that other differential metabolites identified in the present work have been earlier observed at the preclinical stage of the disease. These metabolites include glutamine and BCAAs (leucine and isoleucine), which are reduced in OXYS rats. A link between low levels of BCAAs and AD has been previously reported (Morabito et al., 2014; González-Domínguez et al., 2015; Ikeuchi et al., 2022; Siddik et al., 2022). Some researchers have suggested that decreased level of BCAAs can be considered as a potential marker for predicting the transition from mild cognitive impairment to AD (Nielsen et al., 2021, 2024; Xiong et al., 2022). Yu et al. (2023) suggested that the concentration of BCAAs in the blood of AD patients is significantly reduced because brain cells use the carbon skeleton of BCAAs as an auxiliary fuel to support the impaired energy metabolism in Alzheimer’s disease. However, another randomized study found elevated BCAAs concentrations in the serum of patients with Alzheimer’s disease (Li et al., 2018). These largely opposite results are most likely due to the fact that the BCAAs concentration in blood often directly reflects dietary consumption levels, as they are mainly poorly metabolized in the liver (Griffin and Bradshaw, 2017). Thus, decreased blood concentrations of essential amino acids may indicate underlying nutritional deficiencies in preclinical human dementia (Sato et al., 2021). BCAAs and glutamine are closely related to glutamate metabolism. Glutamine is converted to glutamate, acting as the main excitatory neurotransmitter in the CNS (Chaudhry et al., 2002). As noted in Nielsen’s work (Nielsen et al., 2021), a decrease in BCAA levels can affect the conversion of glutamine and glutamate, and thereby lead to decreased neurotransmission. This is supported by the finding of lower glutamine levels in people with cognitive impairment, as well as in a study of patients with AD, which also reported decreased glutamate levels (Fayed et al., 2011). Glutamine and BCAAs contribute to the Krebs cycle (Dong and Brewer, 2019). Therefore, changes in glutamine and BCAA levels will also affect the functioning of the TCA cycle. Although decreased serum leucine and isoleucine levels were only detected in OXYS rats at 20 days of age, we found decreased levels of the downstream BCAA metabolite 3-methyl-2-oxavalerate during the manifestation and progression of AD. We suggest that the decreased level of 3-methyl-2-oxavalerate may indicate BCAA dysregulation, which in turn may be a cause and/or consequence of mitochondrial dysfunction (Menni et al., 2013). Indeed, we have previously shown that the development of AD signs in OXYS rats occurs against the background of mitochondrial dysfunction from an early age (Kolosova et al., 2022). It can be assumed that the decrease in the level of 3-methyl-2-oxavalerate in OXYS rats reflects disturbances in mitochondrial metabolism at the early and late stages of the development of AD-like degeneration. Thus, the set of differential metabolites found in young OXYS versus Wistar rats is similar to that in AD patients versus healthy controls, and the same metabolic pathways are affected. Differential metabolites found in the blood of 20-day-old rats can be considered as potential early biomarkers of neurodegeneration.

According to our data, the only metabolite that differed in OXYS compared to Wistar rats at all stages of AD-like pathology development was pyruvate, the end product of glycolysis and a substrate for the synthesis of mitochondrial adenosine triphosphate. Decreased level of pyruvate was observed in the blood of patients with mild to moderate AD (Nielsen et al., 2024), but increased pyruvate levels were observed in CSF of patients with AD (Parnetti et al., 1995). However, we believe that changes in pyruvate levels reflect disturbances in energy metabolism that are characteristic of the development of neurodegenerative diseases in human and OXYS rats. This is also supported by data obtained from patients with Parkinson’s disease (decreased pyruvate levels were found in blood samples) (Ahmed et al., 2009), which makes it questionable to use pyruvate as a specific biomarker of AD.

A common feature of old (16 months) OXYS rats and humans with developed AD is mitochondrial dysfunction (Tyumentsev et al., 2018), which alters the energy metabolism. Indeed, the differential metabolites and the most affected pathways found in 16-month-old OXYS rats compared to Wistar rats are mainly related to energy metabolism. These pathways include glycolysis, pyruvate metabolism, and BCAAs biosynthesis and degradation (Figure 3). Other metabolites whose concentrations differ significantly from the control group are threonine, ornithine and 2-ketoisovalerate. The concentrations of these metabolites in the serum of 16-month-old OXYS rats are significantly lower. A similar observation was reported in works (Ozaki et al., 2022; Berezhnoy et al., 2023); ornithine and threonine levels in the serum of patients with AD were reduced. Interestingly, we found high levels of ascorbate (vitamin C in humans) in the serum of aged OXYS rats. In humans, the association between vitamin C and Alzheimer’s disease has been reported to be controversial (Ide et al., 2016; Ulstein and Bøhmer, 2016; Lanyau-Domínguez et al., 2020; Hamid et al., 2022; Appiah et al., 2024). For example, a recent study shows that extremely high serum ascorbate concentrations above 2.3 mg/dL correlates with a higher risk of AD in humans (Appiah et al., 2024). Unlike humans and other primates, mice and rats are able to synthesize ascorbate from d-glucuronate in liver (Gabbay et al., 2010). Unfortunately, we have to note that the exact mechanism that could explain the association between very high ascorbate levels and Alzheimer’s disease progression is unknown.

In our recent paper (Snytnikova et al., 2024), we analyzed the metabolomic differences between the hippocampi of OXYS and Wistar rats. It is interesting to compare the metabolomic changes characteristic of the blood and brain of rats. It should be noted that the brain samples in the work (Snytnikova et al., 2024) were collected within 3 min after death. Therefore, significant metabolomic changes could have occurred due to rapid anaerobic reactions in the brain (Dienel, 2021; Fomenko et al., 2022). However, these changes concern mainly energetic metabolites (such as ATP, ADP, AMP, glucose and lactate) and Krebs cycle compounds. For other metabolites, the influence of postmortem processes is less pronounced (Fomenko et al., 2022). Considering that the postmortem intervals for all animals in the work (Snytnikova et al., 2024) were approximately equal, the data presented for non-energetic compounds of the hippocampus can be used, at least, for qualitative comparison with the data obtained for the blood metabolome in the present work. The comparison shows that some differential metabolites and affected pathways are common for blood and hippocampus, including “Purine metabolism” and “Glyoxylate and dicarboxylate metabolism” for 20-day-old rats, “Pyrimidine metabolism” for 4-month-old rats, and “Valine, leucine and isoleucine biosynthesis” in 16-month-old animals. However, the majority of differential metabolites in the blood and brain differ, and the metabolomic changes in the blood and brain are often in opposite directions. Such contradirectional behavior is observed for several other metabolites, including acetate, cytidine, histidine, and isoleucine. The comparison of age-related changes in the blood and brain of OXYS and Wistar rats for all detected metabolites is presented in Supplementary Figure S4. Therefore, it is obvious that both metabolomic age-related changes and changes induced by neurodegenerative processes in the brain and blood are different.

The development of blood-based biomarkers for Alzheimer’s disease faces challenges such as the unknown ability of the molecule to cross the blood–brain barrier (BBB) and the lack of direct association of peripheral markers with brain processes (Enche Ady et al., 2017). It is believed that BBB disruption occurring with aging play a key role in the early stages of AD (Yu et al., 2024). Moreover, with increasing cognitive impairment, BBB disruption increases, implying an increased relationship between blood and brain metabolite concentrations (Henriksen et al., 2014). Thus, the observed opposite behavior for a number of metabolites found in this study can be explained by the function of the BBB. Indeed, we have previously found altered BBB permeability in the hippocampus in OXYS rats during the first 3 weeks of life (Rudnitskaya et al., 2023). In the present study, we found that in 20-day-old OXYS rats (that corresponds the presymptomatic stage of AD), levels of two metabolites, allantoin and histidine, were increased in serum and decreased in the hippocampus (Supplementary Figure S4). These metabolites are associated with oxidative stress and energy metabolism disorders and are altered in the serum and urine of Alzheimer’s disease patients (Berezhnoy et al., 2023; Yin et al., 2023; Nielsen et al., 2024). In late stage Alzheimer’s disease, we observed decreased threonine levels in both the blood and hippocampus of aged OXYS rats, and we suggest that this metabolite could potentially be considered as a biomarker. Thus, extensive studies have identified metabolite and pathway changes specific to dementia and prediagnostic dementia, in particular altered threonine catabolism in the prediagnostic stage, which extends to several threonine-related pathways in the dementia stage (Figueira et al., 2019).

## 5 Limitations of this study

In this study, we measured metabolomic composition of rat serum and compared it with the metabolomic profile of rat hippocampus reported in Snytnikova et al. (2024). In both studies, animals were anesthetized by asphyxiation with 100% CO_2_ followed by decapitation. It has been noted (Overmyer et al., 2015) that anesthesia by CO_2_ can induce tissue-specific changes in the metabolome due to anaerobic reactions. Therefore, the quantitative data obtained in the present study may be dependent on the method of sacrifice.

## 6 Conclusion

In this study, we investigated the changes in blood metabolites during development of AD signs in OXYS rats. Noteworthy, the most significant changes of the metabolome were found in 20-day-old OXYS rats compared to control Wistar rats, which corresponds to the preclinical period of AD development in humans. Metabolomic changes observed in the brain and blood are different and often opposite in sign, but the presence of common differential metabolites suggests that blood serum is potentially promising fluid for the diagnosis of AD.

## Data Availability

The datasets presented in this study can be found in online repositories. Raw NMR spectra, description of samples and metabolite concentrations are available at the Animal Metabolite Database repository, Experiment ID 299 (https://amdb.online/amdb/experiments/299/, accessed on 29 May 2025). All obtained data are available from the corresponding author upon request.

## Footnotes

^1^ https://www.rscf.ru/

^1^http://www.hmdb.ca (Accessed December 12, 2023).

^1^www.metaboanalyst.ca (Accessed February 12, 2024).

^1^https://colab.google (Accessed December 12, 2023).

^1^https://docs.scipy.org (Accessed December 12, 2023).

^1^https://matplotlib.org (Accessed December 12, 2023).
