# Supplementary material for: Quantitative analysis of serum metabolites in a rat model of Alzheimer’s disease
Source: Front Aging Neurosci. 2025 Sep 23;17:1648561. doi: 10.3389/fnagi.2025.1648561 (PMC12500553; doi:10.3389/fnagi.2025.1648561)
Supplement: Supplementary file 1 [file Supplementary_file_1.DOCX]

**Quantitative Analysis of Serum Metabolites in a Rat Model of Alzheimer's Disease**

Anton A. Smolentsev^1,3,†^, Darya V. Telegina^2,†^, Nataliya G. Kolosova^2^, Yuri P. Tsentalovich^1^, Olga A. Snytnikova^1,^*

^1^*International Tomography Center, Siberian Branch of the Russian Academy of Sciences, Novosibirsk, Russia*

^2^*The Federal Research Center Institute of Cytology and Genetics, Siberian Branch of the Russian Academy of Sciences, Novosibirsk, Russia*

^3^*Novosibirsk State University, Novosibirsk, Russia*

*Correspondence: Olga Snytnikova,

snytnikova_olga@tomo.nsc.ru.

^†^These authors contributed equally to this work.

**Supplementary Information**

**Content:**

**Table S1.** Concentrations of metabolites (µM) in the blood serum of Wistar rats at different ages.

**Table S2.** Concentrations of metabolites (µM) in the blood serum of OXYS rats at different ages.

**Table S3.** One-way analysis of variance of serum metabolite concentrations in OXYS and Wistar rats of different ages. The criterion for significance with *p*-value < 0.05 is indicated in red.

**Table S4.** Two-way analysis of variance of serum metabolite concentrations in OXYS and Wistar rats of different ages. The criterion for significance with *p*-value < 0.05 is indicated in red.

**Table S5.** Result from Quantitative Enrichment Analysis of metabolite sets for serum metabolites from age-matched Wistar and OXYS rats at age of 20 days.

**Table S6.** Result from Quantitative Enrichment Analysis of metabolite sets for serum metabolites from age-matched Wistar and OXYS rats at age of 4 months.

**Table S7.** Result from Quantitative Enrichment Analysis of metabolite sets for serum metabolites from age-matched Wistar and OXYS rats at age of 16 months.

**Figure S1.** Discriminant analysis (sPLS-DA plots) of metabolomic profile of blood serum from Wistar and OXYS rats of various ages.

**Figure S2.** Changes in the concentrations of metabolites in the blood serum of Wistar and OXYS rats depending on age.

**Figure S3.1.** PCA score plot (A), Enriched metabolite sets (B) and Volcano plot (C) for blood serum from Wistar rats at age of 20 days and 4 months.

**Figure S3.2.** PCA score plot (A), Enriched metabolite sets (B) and Volcano plot (C) for blood serum from OXYS rats at age of 20 days and 4 months.

**Figure S3.3.** PCA score plot (A), Enriched metabolite sets (B) and Volcano plot (C) for blood serum from Wistar rats at age of 4 and 16 months.

**Figure S3.4.** PCA score plot (A), Enriched metabolite sets (B) and Volcano plot (C) for blood serum from OXYS rats at age of 4 and 16 months.

**Figure** **S4**. Changes in the concentrations of metabolites in the blood serum and hippocampus of Wistar and OXYS rats at different stages of AD: presymptomatic (at 20 days of age), early (3-4 months) and late (16-18 months).

**Table S1.** Concentrations of metabolites (µM) in the blood serum of Wistar rats at different ages.

| **Metabolite** | **20 days** | | **4 months** | | **16 months** | |
| --- | --- | --- | --- | --- | --- | --- |
|  | **N = 6** | | **N = 6** | | **N = 6** | |
|  | **Min - max** | **Mean ± sd** | **Min - max** | **Mean ± sd** | **Min - max** | **Mean ± sd** |
| 2-Ketoisovalerate | 0.2 - 7.3 | 4.1 ± 2.5 | 3.3 - 5.8 | 4.4 ± 0.9 | 6.1 - 8.5 | 7.9 ± 0.9 |
| 3-Hydroxybutyrate | 83 - 930 | 300 ± 300 | 50 - 185 | 90 ± 50 | 42 - 111 | 67 ± 26 |
| 3-Methyl-2-oxovalerate | 7.5 - 19.6 | 14 ± 5 | 8.8 - 12.8 | 10.6 ± 1.8 | 9.1 - 20.1 | 15 ± 4 |
| Acetate | 126 - 240 | 200 ± 40 | 41 - 99 | 72 ± 23 | 62 - 95 | 79 ± 14 |
| Acetoacetate | 3 - 27 | 14 ± 9 | 7.8 - 28.4 | 14 ± 8 | 16 - 37 | 22 ± 7 |
| Acetone | 8 - 52 | 25 ± 17 | 2.3 - 12.6 | 5 ± 4 | 0.8 - 5.9 | 3.4 ± 2.2 |
| Acetylcarnitine | 8.2 - 12.9 | 11.2 ± 1.8 | 16 - 31 | 21 ± 5 | 15 - 23 | 18.7 ± 3.0 |
| Alanine | 503 - 867 | 690 ± 180 | 463 - 542 | 500 ± 30 | 403 - 713 | 580 ± 100 |
| Allantoin | 32 - 52 | 39 ± 9 | 59 - 85 | 73 ± 10 | 42 - 59 | 50 ± 7 |
| alpha-Aminobutyrate | 6.6 - 14.1 | 10.4 ± 2.9 | 2.3 - 10.6 | 6.9 ± 2.8 | 3.0 - 6.8 | 5.1 ± 1.5 |
| Arginine | 44 - 87 | 63 ± 18 | 8.9 - 135.6 | 60 ± 50 | 97 - 284 | 190 ± 80 |
| Ascorbate | 0.3 - 1.6 | 0.9 ± 0.4 | 6.7 - 28.8 | 19 ± 7 | 2.6 - 10.9 | 5.8 ± 2.9 |
| Asparagine | 76 - 124 | 105 ± 20 | 48 - 88 | 71 ± 13 | 79 - 110 | 90 ± 11 |
| Aspartate | 11 - 59 | 34 ± 16 | 44 - 53 | 48 ± 4 | 30 - 74 | 54 ± 16 |
| Betaine | 111 - 238 | 190 ± 50 | 150 - 193 | 174 ± 14 | 81 - 167 | 130 ± 30 |
| Carnitine | 19 - 32 | 24 ± 6 | 36 - 66 | 52 ± 11 | 53 - 62 | 56 ± 3 |
| Choline | 9.7 - 18.8 | 14 ± 4 | 15 - 27 | 23 ± 5 | 16 - 22 | 18.7 ± 2.2 |
| Citrate | 202 - 334 | 280 ± 40 | 204 - 272 | 238 ± 28 | 128 - 264 | 210 ± 50 |
| Creatine | 130 - 216 | 180 ± 30 | 256 - 331 | 296 ± 30 | 318 - 415 | 350 ± 40 |
| Creatinine | 14 - 27 | 22 ± 5 | 20 - 31 | 26 ± 4 | 28 - 37 | 32 ± 4 |
| Cytidine | 22 - 46 | 35 ± 8 | 60 - 80 | 68 ± 8 | 51 - 69 | 60 ± 7 |
| Deoxycytidine | 14 - 60 | 41 ± 20 | 51 - 69 | 61 ± 7 | 39 - 64 | 52 ± 10 |
| Dimethylamine | 0.3 - 1.8 | 1.2 ± 0.7 | 0.1 - 2.3 | 0.9 ± 0.9 | 0.3 - 0.7 | 0.56 ± 0.13 |
| Dimethylglycine | 8.6 - 13.8 | 10.3 ± 2.0 | 0.3 - 2.6 | 1.6 ± 1 | 3.0 - 4.3 | 3.8 ± 0.4 |
| Formate | 31 - 58 | 39 ± 10 | 21 - 38 | 29 ± 7 | 21 - 32 | 24 ± 4 |
| Fumarate | 0.3 - 3.4 | 1.7 ± 1.3 | 1.6 - 5.8 | 2.7 ± 1.6 | 1.1 - 1.7 | 1.42 ± 0.18 |
| Glucose | 1925 - 2536 | 2190 ± 280 | 2306 - 4200 | 3200 ± 800 | 2187 - 3401 | 2700 ± 400 |
| Glutamate | 151 - 258 | 210 ± 40 | 195 - 301 | 240 ± 50 | 157 - 267 | 210 ± 40 |
| Glutamine | 486 - 675 | 600 ± 70 | 486 - 642 | 550 ± 70 | 603 - 869 | 750 ± 90 |
| Glycerol | 101 - 162 | 135 ± 22 | 70 - 171 | 120 ± 40 | 98 - 182 | 143 ± 29 |
| Glycine | 196 - 238 | 219 ± 16 | 254 - 342 | 290 ± 40 | 262 - 386 | 330 ± 50 |
| Histidine | 37 - 58 | 45 ± 7 | 48 - 78 | 60 ± 11 | 58 - 83 | 68 ± 9 |
| Isobutyrate | 13 - 20 | 17.0 ± 2.3 | 9.1 - 13.1 | 10.4 ± 1.5 | 10 - 13 | 11.5 ± 1.1 |
| Isoleucine | 68 - 98 | 84 ± 13 | 72 - 105 | 82 ± 13 | 79 - 120 | 101 ± 15 |
| Ketoleucine | 3.4 - 13.2 | 8 ± 4 | 2.0 - 6.1 | 4.2 ± 1.7 | 3.8 - 9.9 | 7.3 ± 2.1 |
| Lactate | 2364 - 6966 | 4100 ± 1800 | 3264 - 6748 | 5100 ± 1200 | 3951 - 5159 | 4400 ± 400 |
| Leucine | 92 - 175 | 140 ± 30 | 109 - 171 | 133 ± 21 | 105 - 172 | 151 ± 23 |
| Lysine | 101 - 160 | 127 ± 25 | 203 - 292 | 240 ± 40 | 278 - 372 | 310 ± 30 |
| Mannose | 19 - 26 | 22.5 ± 2.6 | 22 - 50 | 33 ± 10 | 24 - 34 | 30 ± 3 |
| Methionine | 4.1 - 4.7 | 4.39 ± 0.19 | 51 - 72 | 64 ± 7 | 68 - 109 | 87 ± 15 |
| Methionine sulfoxide | 30 - 63 | 46 ± 14 | 1.7 - 3.3 | 2.8 ± 0.6 | 3.3 - 4.9 | 3.9 ± 0.6 |
| Ornithine | 50 - 92 | 68 ± 15 | 28 - 36 | 32 ± 3 | 34 - 49 | 41 ± 6 |
| Pantothenate | 0.7 - 3.1 | 2.3 ± 1.0 | 1.0 - 2.3 | 1.4 ± 0.5 | 0.9 - 1.7 | 1.28 ± 0.27 |
| Phenylalanine | 30 - 89 | 61 ± 23 | 62 - 87 | 70 ± 9 | 62 - 89 | 73 ± 10 |
| Phosphocholine | 2.4 - 4.3 | 3.1 ± 0.7 | 3.3 - 6.2 | 4.6 ± 1.1 | 1.1 - 3.9 | 2.2 ± 1.1 |
| Proline | 115 - 293 | 180 ± 70 | 123 - 196 | 164 ± 29 | 130 - 225 | 190 ± 40 |
| Pyruvate | 7 - 37 | 26 ± 13 | 202 - 313 | 240 ± 50 | 138 - 385 | 280 ± 90 |
| Sarcosine | 1.6 - 7.1 | 4.2 ± 1.9 | 1.7 - 2.2 | 1.97 ± 0.19 | 2.0 - 3.4 | 2.7 ± 0.5 |
| scyllo-Inositol | 75 - 128 | 105 ± 22 | 41 - 84 | 63 ± 16 | 36 - 51 | 42 ± 6 |
| Serine | 172 - 274 | 210 ± 40 | 153 - 205 | 182 ± 20 | 164 - 260 | 220 ± 40 |
| Succinate | 18 - 79 | 43 ± 24 | 27 - 190 | 60 ± 60 | 25 - 49 | 35 ± 9 |
| Threonine | 113 - 258 | 160 ± 50 | 195 - 306 | 230 ± 40 | 248 - 463 | 350 ± 90 |
| Tryptophan | 52 - 93 | 71 ± 17 | 76 - 91 | 82 ± 5 | 55 - 92 | 81 ± 14 |
| Tyrosine | 64 - 156 | 100 ± 30 | 63 - 103 | 79 ± 13 | 68 - 97 | 83 ± 12 |
| Valine | 110 - 187 | 150 ± 30 | 150 - 208 | 170 ± 22 | 173 - 241 | 213 ± 23 |

**Table S2.** Concentrations of metabolites (µM) in the blood serum of OXYS rats at different ages.

| **Metabolite** | **20 days** | | **4 months** | | **16 months** | |
| --- | --- | --- | --- | --- | --- | --- |
|  | **N = 7** | | **N = 6** | | **N = 6** | |
|  | **Min - max** | **Mean ± sd** | **Min - max** | **Mean ± sd** | **Min - max** | **Mean ± sd** |
| 2-Ketoisovalerate | 4.5 - 7.5 | 5.6 ± 1.0 | 2.4 - 5.0 | 4.0 ± 0.9 | 3.2 - 6.6 | 4.9 ± 1.3 |
| 3-Hydroxybutyrate | 133 - 585 | 350 ± 180 | 67 - 124 | 89 ± 21 | 57 - 111 | 80 ± 22 |
| 3-Methyl-2-oxovalerate | 7.9 - 14.6 | 11.2 ± 2.3 | 5.4 - 8.9 | 7.1 ± 1.2 | 4.3 - 11.5 | 7.4 ± 2.5 |
| Acetate | 148 - 212 | 185 ± 23 | 61 - 158 | 90 ± 40 | 81 - 109 | 92 ± 10 |
| Acetoacetate | 3.1 - 19.1 | 11 ± 7 | 9.7 - 20.3 | 14 ± 4 | 16 - 23 | 19.2 ± 2.6 |
| Acetone | 8.7 - 41.1 | 24 ± 12 | 2.4 - 6.7 | 4.7 ± 1.5 | 2.2 - 7.9 | 4.9 ± 2.0 |
| Acetylcarnitine | 8.6 - 12.9 | 11.1 ± 1.7 | 18 - 24 | 20.5 ± 2.4 | 18 - 24 | 20.4 ± 2.2 |
| Alanine | 466 - 673 | 600 ± 70 | 396 - 540 | 460 ± 50 | 367 - 620 | 500 ± 90 |
| Allantoin | 44 - 66 | 52 ± 7 | 57 - 73 | 64 ± 5 | 42 - 58 | 51 ± 6 |
| alpha-Aminobutyrate | 7.6 - 10.5 | 9 ± 1 | 2.4 - 10.0 | 5.7 ± 2.8 | 3.8 - 6.8 | 5.2 ± 1.3 |
| Arginine | 64 - 91 | 75 ± 12 | 4.9 - 132.5 | 70 ± 50 | 54 - 254 | 150 ± 90 |
| Ascorbate | 0.9 - 2.0 | 1.4 ± 0.4 | 13 - 32 | 20 ± 7 | 13 - 19 | 15.9 ± 2.0 |
| Asparagine | 96 - 120 | 112 ± 8 | 58 - 84 | 70 ± 11 | 56 - 104 | 83 ± 16 |
| Aspartate | 26 - 40 | 34 ± 5 | 42 - 70 | 54 ± 12 | 27 - 75 | 50 ± 21 |
| Betaine | 119 - 298 | 170 ± 60 | 105 - 172 | 139 ± 25 | 109 - 178 | 132 ± 27 |
| Carnitine | 23 - 29 | 25.9 ± 1.8 | 39 - 58 | 50 ± 8 | 48 - 58 | 53 ± 4 |
| Choline | 7.4 - 17.1 | 11 ± 4 | 20 - 21 | 20.6 ± 0.4 | 11 - 25 | 19 ± 5 |
| Citrate | 223 - 320 | 270 ± 40 | 146 - 192 | 174 ± 21 | 122 - 225 | 180 ± 40 |
| Creatine | 248 - 287 | 264 ± 15 | 278.7 - 407.3 | 330 ± 40 | 307 - 439 | 360 ± 50 |
| Creatinine | 21 - 30 | 25 ± 3 | 22 - 28 | 25.2 ± 2.6 | 26 - 37 | 31 ± 4 |
| Cytidine | 23 - 51 | 35 ± 12 | 56 - 60 | 58.0 ± 1.5 | 53 - 66 | 61 ± 6 |
| Deoxycytidine | 15 - 53 | 33 ± 13 | 53 - 57 | 54.9 ± 2.0 | 48 - 65 | 56 ± 6 |
| Dimethylamine | 0.8 - 1.8 | 1.3 ± 0.4 | 0.5 - 2.8 | 1.9 ± 1.1 | 0.5 - 1.3 | 0.9 ± 0.3 |
| Dimethylglycine | 8.1 - 10.9 | 9.5 ± 1.3 | 0.3 - 2.5 | 1.8 ± 0.9 | 2.9 - 4.8 | 3.6 ± 0.7 |
| Formate | 35 - 218 | 80 ± 70 | 19 - 42 | 27 ± 9 | 19 - 36 | 30 ± 6 |
| Fumarate | 0.5 - 2.4 | 1.2 ± 0.7 | 1.0 - 2.4 | 1.6 ± 0.6 | 0.5 - 1.9 | 1.2 ± 0.5 |
| Glucose | 2201 - 2706 | 2430 ± 200 | 2628 - 4514 | 3300 ± 700 | 2726 - 3354 | 3000 ± 270 |
| Glutamate | 136 - 204 | 179 ± 21 | 165 - 206 | 186 ± 17 | 132 - 253 | 200 ± 40 |
| Glutamine | 402 - 572 | 490 ± 60 | 467 - 580 | 540 ± 50 | 578 - 735 | 670 ± 60 |
| Glycerol | 123 - 209 | 160 ± 30 | 64 - 221 | 120 ± 70 | 60 - 95 | 77 ± 16 |
| Glycine | 217 - 332 | 280 ± 40 | 282 - 340 | 315 ± 24 | 268 - 342 | 310 ± 30 |
| Histidine | 50 - 67 | 60 ± 7 | 62 - 71 | 67 ± 3 | 59 - 75 | 70 ± 6 |
| Isobutyrate | 8.7 - 18.3 | 15 ± 3 | 8.6 - 11.0 | 10.0 ± 0.8 | 8.2 - 13.8 | 11.1 ± 2.2 |
| Isoleucine | 53 - 84 | 63 ± 10 | 81 - 106 | 88 ± 9 | 75 - 119 | 99 ± 16 |
| Ketoleucine | 5.1 - 11.8 | 7.7 ± 2.2 | 1.7 - 4.3 | 3.3 ± 0.9 | 3.1 - 6.6 | 4.4 ± 1.5 |
| Lactate | 2780 - 7651 | 4500 ± 1700 | 2408 - 4256 | 3600 ± 600 | 3313 - 4963 | 3900 ± 700 |
| Leucine | 85 - 120 | 103 ± 11 | 118 - 190 | 151 ± 25 | 119 - 176 | 151 ± 22 |
| Lysine | 118 - 360 | 250 ± 80 | 208 - 291 | 245 ± 29 | 227 - 327 | 270 ± 40 |
| Mannose | 23 - 33 | 29 ± 4 | 22 - 41 | 31 ± 6 | 26 - 35 | 31 ± 3 |
| Methionine | 4.2 - 4.7 | 4.46 ± 0.19 | 62 - 74 | 66 ± 4 | 51 - 88 | 68 ± 12 |
| Methionine sulfoxide | 32 - 60 | 45 ± 9 | 3.1 - 3.5 | 3.25 ± 0.16 | 3.3 - 5.0 | 4.1 ± 0.7 |
| Ornithine | 57 - 110 | 80 ± 22 | 24 - 41 | 28 ± 7 | 25 - 40 | 33 ± 6 |
| Pantothenate | 1.6 - 3.9 | 2.7 ± 0.8 | 0.9 - 2.7 | 1.5 ± 0.7 | 0.9 - 2.5 | 1.4 ± 0.6 |
| Phenylalanine | 60 - 91 | 75 ± 11 | 59 - 80 | 70 ± 8 | 61 - 72 | 67 ± 4 |
| Phosphocholine | 2.6 - 4.2 | 3.5 ± 0.6 | 2.9 - 4.3 | 3.4 ± 0.6 | 0.7 - 2.8 | 1.6 ± 0.9 |
| Proline | 125 - 182 | 164 ± 19 | 132 - 158 | 140 ± 10 | 102 - 155 | 131 ± 21 |
| Pyruvate | 26 - 71 | 44 ± 20 | 160 - 251 | 200 ± 30 | 123 - 277 | 190 ± 50 |
| Sarcosine | 3.8 - 6.1 | 4.4 ± 0.8 | 2.4 - 3.9 | 3.1 ± 0.6 | 2.1 - 5.3 | 3.4 ± 1.1 |
| scyllo-Inositol | 96 - 136 | 117 ± 15 | 31 - 86 | 59 ± 22 | 36 - 59 | 43 ± 9 |
| Serine | 202 - 342 | 270 ± 50 | 148 - 190.2 | 173 ± 17 | 126 - 230 | 180 ± 40 |
| Succinate | 22 - 44 | 33 ± 9 | 21 - 41 | 31 ± 8 | 16 - 66 | 40 ± 18 |
| Threonine | 154 - 214 | 182 ± 21 | 195 - 238 | 219 ± 18 | 192 - 280 | 210 ± 30 |
| Tryptophan | 71 - 97 | 82 ± 9 | 65 - 96 | 82 ± 12 | 67 - 87 | 78 ± 8 |
| Tyrosine | 98 - 116 | 110 ± 6 | 78 - 91 | 84 ± 5 | 77 - 113 | 94 ± 13 |
| Valine | 113 - 161 | 130 ± 16 | 163 - 214 | 178 ± 19 | 171 - 218 | 201 ± 20 |

**Table S3.** One-way analysis of variance of serum metabolite concentrations in OXYS and Wistar rats of different ages. The significance criterion F at *p*-value < 0.05 is indicated in red.

| \| **Metabolite** \| \| --- \| \| \| \| | **Wistar** | | | | **OXYS** | | | | **OXYS and Wistar** | | | | | |
| --- | --- | --- | --- | --- | --- | --- | --- | --- | --- | --- | --- | --- | --- | --- | --- |
|  | **20d and 4m** | | **4м and 16m** | | **20d and 4m** | | **4м and 16m** | | **20d** | | **4m** | | **16m** | |
|  | **F** | **p** | **F** | **p** | **F** | **p** | **F** | **p** | **F** | **p** | **F** | **p** | **F** | **p** |
| 2-Ketoisovalerate | 0.08 | 0.78 | 47.16 | 0.00 | 9.19 | 0.01 | 1.88 | 0.20 | 2.01 | 0.18 | 0.81 | 0.39 | 22.20 | 0.00 |
| 3-Hydroxybutyrate | 2.79 | 0.13 | 0.84 | 0.38 | 11.3 | 0.01 | 0.59 | 0.46 | 0.07 | 0.80 | 0.00 | 0.98 | 0.81 | 0.39 |
| 3-Methyl-2-oxovalerate | 2.40 | 0.15 | 6.85 | 0.03 | 16.4 | 0.00 | 0.06 | 0.81 | 1.56 | 0.24 | 16.21 | 0.00 | 16.93 | 0.00 |
| Acetate | 40.4 | 0.00 | 0.40 | 0.54 | 33.5 | 0.00 | 0.11 | 0.74 | 0.51 | 0.49 | 0.62 | 0.45 | 3.23 | 0.10 |
| Acetoacetate | 0.04 | 0.85 | 3.35 | 0.10 | 0.71 | 0.42 | 6.52 | 0.03 | 0.28 | 0.61 | 0.02 | 0.88 | 1.02 | 0.34 |
| Acetone | 7.63 | 0.02 | 0.49 | 0.50 | 14.1 | 0.00 | 0.04 | 0.85 | 0.01 | 0.94 | 0.00 | 0.99 | 1.52 | 0.25 |
| Acetylcarnitine | 18.8 | 0.00 | 0.91 | 0.36 | 71.1 | 0.00 | 0.02 | 0.90 | 0.02 | 0.90 | 0.06 | 0.82 | 1.15 | 0.31 |
| Alanine | 6.7 | 0.03 | 3.01 | 0.11 | 15.4 | 0.00 | 0.54 | 0.48 | 1.38 | 0.26 | 1.94 | 0.19 | 2.16 | 0.17 |
| Allantoin | 40.9 | 0.00 | 21.15 | 0.00 | 11.6 | 0.01 | 15.44 | 0.00 | 8.46 | 0.01 | 3.67 | 0.08 | 0.12 | 0.74 |
| alpha-Aminobutyrate | 4.8 | 0.05 | 1.95 | 0.19 | 9.65 | 0.01 | 0.15 | 0.71 | 1.19 | 0.30 | 0.56 | 0.47 | 0.02 | 0.90 |
| Arginine | 0.01 | 0.93 | 11.20 | 0.01 | 0.09 | 0.78 | 3.71 | 0.08 | 2.05 | 0.18 | 0.02 | 0.90 | 0.62 | 0.45 |
| Ascorbate | 37.4 | 0.00 | 16.94 | 0.00 | 58.5 | 0.00 | 2.41 | 0.15 | 3.33 | 0.10 | 0.17 | 0.69 | 50.06 | 0.00 |
| Asparagine | 12.4 | 0.01 | 7.79 | 0.02 | 62.5 | 0.00 | 2.74 | 0.13 | 0.59 | 0.46 | 0.01 | 0.91 | 0.73 | 0.41 |
| Aspartate | 4.64 | 0.06 | 0.82 | 0.39 | 16.5 | 0.00 | 0.15 | 0.71 | 0.00 | 0.99 | 1.24 | 0.29 | 0.16 | 0.70 |
| Betaine | 0.44 | 0.52 | 8.72 | 0.01 | 1.71 | 0.22 | 0.23 | 0.64 | 0.20 | 0.66 | 8.32 | 0.02 | 0.00 | 0.97 |
| Carnitine | 30.6 | 0.00 | 0.59 | 0.46 | 62.6 | 0.00 | 0.55 | 0.48 | 0.42 | 0.53 | 0.20 | 0.67 | 2.52 | 0.14 |
| Choline | 13.7 | 0.00 | 5.09 | 0.05 | 33.0 | 0.00 | 0.53 | 0.49 | 1.36 | 0.27 | 2.16 | 0.17 | 0.05 | 0.83 |
| Citrate | 4.31 | 0.07 | 0.98 | 0.35 | 35.2 | 0.00 | 0.07 | 0.79 | 0.19 | 0.67 | 20.54 | 0.00 | 1.86 | 0.20 |
| Creatine | 41.1 | 0.00 | 8.68 | 0.02 | 13.7 | 0.00 | 1.78 | 0.21 | 36.53 | 0.00 | 2.30 | 0.16 | 0.27 | 0.62 |
| Creatinine | 2.38 | 0.15 | 7.41 | 0.02 | 0.00 | 0.96 | 8.30 | 0.02 | 2.78 | 0.12 | 0.05 | 0.83 | 0.38 | 0.55 |
| Cytidine | 50.8 | 0.00 | 4.22 | 0.07 | 21.7 | 0.00 | 1.44 | 0.26 | 0.00 | 1.00 | 10.47 | 0.01 | 0.13 | 0.72 |
| Deoxycytidine | 5.35 | 0.04 | 3.74 | 0.08 | 16.3 | 0.00 | 0.14 | 0.72 | 0.72 | 0.41 | 4.80 | 0.05 | 0.74 | 0.41 |
| Dimethylamine | 0.61 | 0.45 | 0.92 | 0.36 | 1.48 | 0.25 | 4.59 | 0.06 | 0.10 | 0.76 | 2.93 | 0.12 | 5.94 | 0.04 |
| Dimethylglycine | 93.3 | 0.00 | 25.15 | 0.00 | 147 | 0.00 | 14.57 | 0.00 | 0.88 | 0.37 | 0.27 | 0.62 | 0.35 | 0.57 |
| Formate | 3.59 | 0.09 | 1.92 | 0.20 | 3.58 | 0.09 | 0.55 | 0.48 | 2.17 | 0.17 | 0.25 | 0.63 | 3.37 | 0.10 |
| Fumarate | 1.38 | 0.27 | 3.72 | 0.08 | 0.82 | 0.39 | 1.83 | 0.21 | 0.61 | 0.45 | 2.50 | 0.15 | 1.69 | 0.22 |
| Glucose | 8.10 | 0.02 | 1.51 | 0.25 | 8.72 | 0.01 | 0.76 | 0.40 | 3.55 | 0.09 | 0.06 | 0.82 | 2.03 | 0.19 |
| Glutamate | 2.03 | 0.19 | 1.20 | 0.30 | 0.43 | 0.52 | 0.47 | 0.51 | 1.94 | 0.19 | 7.99 | 0.02 | 0.43 | 0.53 |
| Glutamine | 1.16 | 0.31 | 15.54 | 0.00 | 2.93 | 0.12 | 18.61 | 0.00 | 9.64 | 0.01 | 0.19 | 0.68 | 2.62 | 0.14 |
| Glycerol | 0.88 | 0.37 | 1.55 | 0.24 | 2.50 | 0.14 | 2.08 | 0.18 | 2.83 | 0.12 | 0.00 | 0.99 | 22.70 | 0.00 |
| Glycine | 21.8 | 0.00 | 1.48 | 0.25 | 4.16 | 0.07 | 0.06 | 0.81 | 12.58 | 0.01 | 1.57 | 0.24 | 0.32 | 0.59 |
| Histidine | 7.18 | 0.02 | 2.15 | 0.17 | 6.05 | 0.03 | 0.96 | 0.35 | 14.76 | 0.00 | 2.27 | 0.16 | 0.09 | 0.77 |
| Isobutyrate | 35.7 | 0.00 | 2.18 | 0.17 | 15.5 | 0.00 | 1.38 | 0.27 | 1.25 | 0.29 | 0.45 | 0.52 | 0.20 | 0.66 |
| Isoleucine | 0.05 | 0.83 | 5.52 | 0.04 | 22.9 | 0.00 | 1.85 | 0.20 | 10.50 | 0.01 | 0.94 | 0.35 | 0.06 | 0.81 |
| Ketoleucine | 7.23 | 0.02 | 8.40 | 0.02 | 22.0 | 0.00 | 2.51 | 0.14 | 0.18 | 0.68 | 1.35 | 0.27 | 7.98 | 0.02 |
| Lactate | 1.28 | 0.29 | 1.43 | 0.26 | 1.62 | 0.23 | 0.73 | 0.41 | 0.23 | 0.64 | 6.72 | 0.03 | 1.95 | 0.19 |
| Leucine | 0.18 | 0.68 | 1.95 | 0.19 | 21.4 | 0.00 | 0.00 | 0.99 | 7.95 | 0.02 | 1.79 | 0.21 | 0.00 | 0.99 |
| Lysine | 44.6 | 0.00 | 9.68 | 0.01 | 0.01 | 0.93 | 1.93 | 0.20 | 12.52 | 0.01 | 0.00 | 0.99 | 2.95 | 0.12 |
| Mannose | 6.84 | 0.03 | 0.76 | 0.40 | 0.72 | 0.41 | 0.03 | 0.87 | 12.02 | 0.01 | 0.27 | 0.61 | 0.80 | 0.39 |
| Methionine | 377. | 0.00 | 12.09 | 0.01 | 1616 | 0.00 | 0.08 | 0.79 | 0.56 | 0.47 | 0.66 | 0.44 | 6.23 | 0.03 |
| Methionine sulfoxide | 55.9 | 0.00 | 10.85 | 0.01 | 138 | 0.00 | 9.54 | 0.01 | 0.03 | 0.87 | 3.92 | 0.08 | 0.47 | 0.51 |
| Ornithine | 33.1 | 0.00 | 11.73 | 0.01 | 30.3 | 0.00 | 1.87 | 0.20 | 1.25 | 0.29 | 1.86 | 0.20 | 6.84 | 0.03 |
| Pantothenate | 3.63 | 0.09 | 0.49 | 0.50 | 8.14 | 0.02 | 0.12 | 0.74 | 0.78 | 0.40 | 0.05 | 0.82 | 0.20 | 0.66 |
| Phenylalanine | 0.71 | 0.42 | 0.40 | 0.54 | 0.94 | 0.35 | 0.55 | 0.48 | 1.97 | 0.19 | 0.00 | 0.98 | 2.07 | 0.18 |
| Phosphocholine | 7.33 | 0.02 | 12.60 | 0.01 | 0.05 | 0.84 | 16.69 | 0.00 | 1.52 | 0.24 | 4.52 | 0.06 | 1.00 | 0.34 |
| Proline | 0.33 | 0.58 | 1.36 | 0.27 | 7.72 | 0.02 | 0.89 | 0.37 | 0.40 | 0.54 | 3.54 | 0.09 | 10.36 | 0.01 |
| Pyruvate | 122 | 0.00 | 1.29 | 0.28 | 106 | 0.00 | 0.03 | 0.87 | 3.85 | 0.08 | 3.39 | 0.10 | 4.84 | 0.05 |
| Sarcosine | 8.33 | 0.02 | 11.44 | 0.01 | 11.8 | 0.01 | 0.42 | 0.53 | 0.11 | 0.74 | 19.69 | 0.00 | 1.75 | 0.22 |
| scyllo-Inositol | 13.36 | 0.00 | 9.03 | 0.01 | 31.1 | 0.00 | 2.68 | 0.13 | 1.33 | 0.27 | 0.16 | 0.70 | 0.06 | 0.82 |
| Serine | 1.75 | 0.22 | 3.94 | 0.08 | 21.4 | 0.00 | 0.09 | 0.77 | 6.53 | 0.03 | 0.78 | 0.40 | 3.27 | 0.10 |
| Succinate | 0.36 | 0.56 | 0.87 | 0.37 | 0.09 | 0.77 | 0.98 | 0.35 | 1.02 | 0.33 | 1.13 | 0.31 | 0.31 | 0.59 |
| Threonine | 5.67 | 0.04 | 9.13 | 0.01 | 11.8 | 0.01 | 0.12 | 0.73 | 0.71 | 0.42 | 0.30 | 0.60 | 12.43 | 0.01 |
| Tryptophan | 2.57 | 0.14 | 0.05 | 0.82 | 0.00 | 0.98 | 0.55 | 0.48 | 2.35 | 0.15 | 0.00 | 1.00 | 0.23 | 0.64 |
| Tyrosine | 2.49 | 0.15 | 0.29 | 0.60 | 73.7 | 0.00 | 3.34 | 0.10 | 0.59 | 0.46 | 0.70 | 0.42 | 2.35 | 0.16 |
| Valine | 2.08 | 0.18 | 10.81 | 0.01 | 24.8 | 0.00 | 4.04 | 0.07 | 1.59 | 0.23 | 0.47 | 0.51 | 0.93 | 0.36 |

**Table S4.** Two-way analysis of variance of serum metabolite concentrations in OXYS and Wistar rats of different ages. The significance criterion F at *p*-value < 0.05 is indicated in red.

| **Metabolite** | **Genotype** | | **Age** | | **Genotype * Age** | |
| --- | --- | --- | --- | --- | --- | --- |
|  | **F(1, 36)** | **p** | **F (2, 34)** | **p** | **F (2, 36)** | **p** |
| 2-Ketoisovalerate | 2.008 | 0.166 | 8.059 | 0.002 | 8.505 | 0.001 |
| 3-Hydroxybutyrate | 0.114 | 0.738 | 10.841 | 0.000 | 0.044 | 0.957 |
| 3-Methyl-2-oxovalerate | 22.065 | 0.000 | 5.127 | 0.012 | 2.912 | 0.069 |
| Acetate | 0.203 | 0.656 | 65.394 | 0.000 | 0.986 | 0.384 |
| Acetoacetate | 0.863 | 0.360 | 5.491 | 0.009 | 0.123 | 0.884 |
| Acetone | 0.008 | 0.928 | 20.248 | 0.000 | 0.043 | 0.958 |
| Acetylcarnitine | 0.099 | 0.756 | 40.185 | 0.000 | 0.463 | 0.634 |
| Alanine | 4.433 | 0.043 | 9.083 | 0.001 | 0.237 | 0.790 |
| Allantoin | 0.734 | 0.398 | 31.022 | 0.000 | 6.411 | 0.005 |
| alpha-Aminobutyrate | 1.249 | 0.272 | 15.971 | 0.000 | 0.388 | 0.682 |
| Arginine | 0.138 | 0.713 | 13.242 | 0.000 | 0.686 | 0.511 |
| Ascorbate | 8.525 | 0.006 | 62.210 | 0.000 | 4.961 | 0.014 |
| Asparagine | 0.003 | 0.954 | 24.837 | 0.000 | 0.724 | 0.493 |
| Aspartate | 0.009 | 0.925 | 7.293 | 0.003 | 0.411 | 0.667 |
| Betaine | 1.642 | 0.209 | 4.913 | 0.014 | 0.589 | 0.561 |
| Carnitine | 0.445 | 0.510 | 80.691 | 0.000 | 0.521 | 0.599 |
| Choline | 1.854 | 0.183 | 20.697 | 0.000 | 0.738 | 0.486 |
| Citrate | 8.356 | 0.007 | 17.835 | 0.000 | 1.618 | 0.215 |
| Creatine | 14.543 | 0.001 | 47.854 | 0.000 | 3.457 | 0.044 |
| Creatinine | 0.264 | 0.611 | 14.374 | 0.000 | 1.546 | 0.229 |
| Cytidine | 1.251 | 0.272 | 47.848 | 0.000 | 1.976 | 0.156 |
| Deoxycytidine | 0.861 | 0.361 | 12.499 | 0.000 | 0.984 | 0.385 |
| Dimethylamine | 4.455 | 0.043 | 3.612 | 0.039 | 1.480 | 0.243 |
| Dimethylglycine | 0.513 | 0.479 | 169.212 | 0.000 | 0.757 | 0.478 |
| Formate | 2.389 | 0.132 | 4.785 | 0.015 | 1.819 | 0.179 |
| Fumarate | 3.772 | 0.061 | 2.667 | 0.085 | 0.648 | 0.530 |
| Glucose | 1.637 | 0.210 | 10.805 | 0.000 | 0.100 | 0.905 |
| Glutamate | 7.260 | 0.011 | 1.137 | 0.334 | 0.984 | 0.385 |
| Glutamine | 9.142 | 0.005 | 23.718 | 0.000 | 1.585 | 0.221 |
| Glycerol | 0.881 | 0.355 | 3.913 | 0.031 | 4.787 | 0.015 |
| Glycine | 3.939 | 0.056 | 12.992 | 0.000 | 3.365 | 0.048 |
| Histidine | 10.115 | 0.003 | 15.140 | 0.000 | 2.434 | 0.104 |
| Isobutyrate | 1.792 | 0.190 | 29.478 | 0.000 | 0.416 | 0.663 |
| Isoleucine | 1.946 | 0.173 | 13.725 | 0.000 | 3.574 | 0.040 |
| Ketoleucine | 4.536 | 0.041 | 13.330 | 0.000 | 1.060 | 0.359 |
| Lactate | 1.399 | 0.246 | 0.057 | 0.945 | 1.939 | 0.161 |
| Leucine | 0.823 | 0.371 | 5.902 | 0.007 | 4.530 | 0.019 |
| Lysine | 4.081 | 0.052 | 14.527 | 0.000 | 10.106 | 0.000 |
| Mannose | 1.130 | 0.296 | 4.894 | 0.014 | 1.975 | 0.156 |
| Methionine | 3.805 | 0.060 | 269.203 | 0.000 | 6.339 | 0.005 |
| Methionine sulfoxide | 0.003 | 0.954 | 158.223 | 0.000 | 0.045 | 0.956 |
| Ornithine | 0.000 | 0.990 | 47.742 | 0.000 | 2.447 | 0.103 |
| Pantothenate | 0.935 | 0.341 | 11.402 | 0.000 | 0.268 | 0.766 |
| Phenylalanine | 0.462 | 0.502 | 0.052 | 0.949 | 2.172 | 0.131 |
| Phosphocholine | 2.024 | 0.165 | 17.800 | 0.000 | 2.639 | 0.087 |
| Proline | 7.155 | 0.012 | 1.183 | 0.320 | 0.957 | 0.395 |
| Pyruvate | 5.564 | 0.025 | 65.710 | 0.000 | 4.143 | 0.025 |
| Sarcosine | 4.083 | 0.052 | 10.492 | 0.000 | 0.546 | 0.585 |
| scyllo-Inositol | 0.344 | 0.562 | 24.271 | 0.000 | 0.841 | 0.594 |
| Serine | 0.229 | 0.636 | 9.668 | 0.001 | 6.829 | 0.003 |
| Succinate | 1.321 | 0.259 | 0.307 | 0.738 | 0.948 | 0.398 |
| Threonine | 7.026 | 0.013 | 15.282 | 0.000 | 9.086 | 0.001 |
| Tryptophan | 0.621 | 0.437 | 0.730 | 0.490 | 1.376 | 0.267 |
| Tyrosine | 2.622 | 0.115 | 7.800 | 0.002 | 0.125 | 0.883 |
| Valine | 1.008 | 0.323 | 29.199 | 0.000 | 1.104 | 0.344 |

**Table S5.** Result from Quantitative Enrichment Analysis of metabolite sets for serum metabolites from age-matched Wistar and OXYS rats at age of 20 days.

|  | Total Cmpd | Hits | Statistic Q | Expected Q | Raw  p | Holm  p | FDR |
| --- | --- | --- | --- | --- | --- | --- | --- |
| Purine metabolism | 70 | 2 | 45.09 | 8.33 | 9.91E-04 | 3.96E-02 | 2.11E-02 |
| Glyoxylate and dicarboxylate metabolism | 31 | 7 | 26.54 | 8.33 | 1.22E-03 | 4.74E-02 | 2.11E-02 |
| Arginine and proline metabolism | 36 | 5 | 26.44 | 8.33 | 2.55E-03 | 9.69E-02 | 2.11E-02 |
| Glycine, serine and threonine metabolism | 33 | 9 | 24.51 | 8.33 | 4.35E-03 | 1.61E-01 | 2.11E-02 |
| Lipoic acid metabolism | 28 | 2 | 39.63 | 8.33 | 4.36E-03 | 1.61E-01 | 2.11E-02 |
| Primary bile acid biosynthesis | 46 | 1 | 53.35 | 8.33 | 4.58E-03 | 1.61E-01 | 2.11E-02 |
| Porphyrin metabolism | 31 | 1 | 53.35 | 8.33 | 4.58E-03 | 1.61E-01 | 2.11E-02 |
| Biotin metabolism | 10 | 1 | 53.23 | 8.33 | 4.65E-03 | 1.61E-01 | 2.11E-02 |
| Fructose and mannose metabolism | 20 | 1 | 52.21 | 8.33 | 5.27E-03 | 1.69E-01 | 2.11E-02 |
| Amino sugar and nucleotide sugar metabolism | 42 | 1 | 52.21 | 8.33 | 5.27E-03 | 1.69E-01 | 2.11E-02 |
| Glutathione metabolism | 28 | 2 | 31.78 | 8.33 | 6.53E-03 | 1.96E-01 | 2.37E-02 |
| Nitrogen metabolism | 6 | 1 | 46.71 | 8.33 | 1.00E-02 | 2.90E-01 | 3.34E-02 |
| Histidine metabolism | 16 | 2 | 28.65 | 8.33 | 1.45E-02 | 4.06E-01 | 4.13E-02 |
| beta-Alanine metabolism | 21 | 2 | 28.65 | 8.33 | 1.45E-02 | 4.06E-01 | 4.13E-02 |
| Galactose metabolism | 27 | 3 | 27.28 | 8.33 | 1.55E-02 | 4.06E-01 | 4.13E-02 |
| Lysine degradation | 30 | 2 | 28.43 | 8.33 | 1.93E-02 | 4.82E-01 | 4.82E-02 |
| D-Amino acid metabolism | 15 | 1 | 37.23 | 8.33 | 2.68E-02 | 6.43E-01 | 5.95E-02 |
| Sphingolipid metabolism | 32 | 1 | 37.23 | 8.33 | 2.68E-02 | 6.43E-01 | 5.95E-02 |
| Valine, leucine and isoleucine biosynthesis | 8 | 7 | 19.86 | 8.33 | 3.06E-02 | 6.73E-01 | 6.44E-02 |
| Cysteine and methionine metabolism | 33 | 3 | 22.65 | 8.33 | 3.91E-02 | 8.21E-01 | 7.66E-02 |
| Valine, leucine and isoleucine degradation | 39 | 7 | 19.34 | 8.33 | 4.02E-02 | 8.21E-01 | 7.66E-02 |
| Arginine biosynthesis | 14 | 4 | 18.15 | 8.33 | 4.88E-02 | 9.28E-01 | 8.88E-02 |

**Table S6.** Result from Quantitative Enrichment Analysis of metabolite sets for serum metabolites from age-matched Wistar and OXYS rats at age of 4 months.

|  | Total Cmpd | Hits | Statistic Q | Expected Q | Raw  p | Holm  p | FDR |
| --- | --- | --- | --- | --- | --- | --- | --- |
| Citrate cycle (TCA cycle) | 20 | 3 | 34.24 | 9.09 | 2.43E-03 | 9.73E-02 | 9.73E-02 |
| Glycine, serine and threonine metabolism | 33 | 9 | 22.20 | 9.09 | 1.32E-02 | 5.15E-01 | 2.64E-01 |
| Pyrimidine metabolism | 39 | 3 | 28.47 | 9.09 | 3.24E-02 | 1.00E+00 | 3.50E-01 |
| Alanine, aspartate and glutamate metabolism | 28 | 6 | 19.29 | 9.09 | 3.88E-02 | 1.00E+00 | 3.50E-01 |
| Glyoxylate and dicarboxylate metabolism | 31 | 7 | 17.65 | 9.09 | 4.77E-02 | 1.00E+00 | 3.50E-01 |

**Table S7.** Result from Quantitative Enrichment Analysis of metabolite sets for serum metabolites from age-matched Wistar and OXYS rats at age of 16 months.

|  | Total Cmpd | Hits | Statistic Q | Expected Q | Raw  p | Holm  p | FDR |
| --- | --- | --- | --- | --- | --- | --- | --- |
| Valine, leucine and isoleucine biosynthesis | 8 | 7 | 34.38 | 9.09 | 1.20E-02 | 4.78E-01 | 2.06E-01 |
| Arginine and proline metabolism | 36 | 5 | 26.50 | 9.09 | 1.87E-02 | 7.29E-01 | 2.06E-01 |
| Glycolysis / Gluconeogenesis | 26 | 2 | 28.50 | 9.09 | 2.39E-02 | 9.07E-01 | 2.06E-01 |
| Pyruvate metabolism | 23 | 2 | 28.50 | 9.09 | 2.39E-02 | 9.07E-01 | 2.06E-01 |
| Valine, leucine and isoleucine degradation | 39 | 7 | 27.79 | 9.09 | 2.57E-02 | 9.25E-01 | 2.06E-01 |
| Cysteine and methionine metabolism | 33 | 3 | 31.87 | 9.09 | 3.99E-02 | 1.00E+00 | 2.66E-01 |

**Figure S1.** Discriminant analysis (sPLS-DA plots) of metabolomic profile of blood serum from Wistar and OXYS rats of various ages. The sign 20d in the figure corresponds to the age of 20 days, 4m and 16m - 4 and 16 months, respectively.

**Figure S2.** Changes in the concentrations of metabolites in the blood serum of Wistar (blue circle) and OXYS (red square) rats depending on age. Data are presented as M ± SD. Significant: * - interstrain differences (*p* < 0.05), # - changes compared to the previous age for Wistar (*p* <0.05), ‡- changes compared to the previous age for OXYS (*p* < 0.05).

**Figure S3.1.** PCA score plot (A), Enriched metabolite sets (B) and Volcano plot (C) for blood serum from Wistar rats at age of 20 days and 4 months. In MSEA plot the metabolic pathways with *p*-value < 0.05 are presented. In Volcano plot, the x-axis displays the fold change (FC), the horizontal line depicts the cut-off of FDR – adjusted *p*-value = 0.05; metabolites with fold change threshold (4 months vs. 20 days) = 1.3 are highlighted.

**Figure S3.2.** PCA score plot (A), Enriched metabolite sets (B) and Volcano plot (C) for blood serum from OXYS rats at age of 20 days and 4 months. In MSEA plot the metabolic pathways with *p*-value < 0.05 are presented. In Volcano plot, the x-axis displays the fold change (FC), the horizontal line depicts the cut-off of FDR – adjusted *p*-value = 0.05; metabolites with fold change threshold (4 months vs. 20 days) = 1.3 are highlighted.

**Figure S3.3.** PCA score plot (A), Enriched metabolite sets (B) and Volcano plot (C) for blood serum from Wistar rats at age of 4 and 16 months. In MSEA plot the metabolic pathways with *p*-value < 0.05 are presented. In Volcano plot, the x-axis displays the fold change (FC), the horizontal line depicts the cut-off of FDR – adjusted *p*-value = 0.05; metabolites with fold change threshold (4 months vs. 20 days) = 1.3 are highlighted.

**Figure S3.4.** PCA score plot (A), Enriched metabolite sets (B) and Volcano plot (C) for blood serum from OXYS rats at age of 4 and 16 months. In MSEA plot the metabolic pathways with *p*-value < 0.05 are presented. In Volcano plot, the x-axis displays the fold change (FC), the horizontal line depicts the cut-off of FDR – adjusted *p*-value = 0.05; metabolites with fold change threshold (4 months vs. 20 days) = 1.3 are highlighted.

**Figure** **S4**. Changes in the concentrations of metabolites in the blood serum and hippocampus of Wistar (blue circle) and OXYS (red square) rats at different stages of AD: presymptomatic (at 20 days of age), early (3-4 months) and late (16-18 months). Data are presented as mean ± SD. Significant differences: **p-*value ˂ 0.05 between rat strains.
